# Effect of topical fluoride applications on residual monomer release from resin-based restorative materials

**DOI:** 10.1186/s12903-022-02698-x

**Published:** 2023-01-02

**Authors:** Ebru Delikan, Ayse Tugba Erturk-Avunduk, Ozcan Karatas, Şerife Saçmacı

**Affiliations:** 1 Department of Pediatric Dentistry, Faculty of Dentistry, Nuh Naci Yazgan University, TR-38170 Kayseri, Türkiye; 2 Department of Restorative Dentistry, Faculty of Dentistry, Mersin University, TR-33343 Mersin, Türkiye; 3 Department of Restorative Dentistry, Faculty of Dentistry, Nuh Naci Yazgan University, TR-38170 Kayseri, Türkiye; 4 Department of Chemistry, Faculty of Sciences, Erciyes University, TR-38039 Kayseri, Türkiye

**Keywords:** Bulk-fill composite resin, Compomer, Fluoride application, HPLC, Monomer release

## Abstract

**Background:**

The effects of topical fluoride applications on the release of monomer ingredients from resin-based dental materials by immersion in various extraction solutions are unclear. The aim of this study was to determine the effect of topical fluorides (APF and NaF) on the elution of residual monomers (Bis-GMA, TEGDMA, UDMA, and HEMA) from resin-based materials.

**Methods:**

Ninety specimens were prepared, 30 bulk-fill composite resin, 30 nanohybrid universal composite resin, and 30 polyacid-modified composite resin (compomer). These were randomly divided into three groups based on fluoride application procedures. Each specimen was kept in 75% ethanol solution, and residual monomers released from materials were analyzed using high-performance liquid chromatography (HPLC) after 10 min, 1 h, 24 h, and 30 days. The groups were compared using the Mann Whitney U and Kruskal Wallis tests. Measurements were analyzed using the Friedman and Wilcoxon signed-rank tests.

**Results:**

Fluoride applications generally had no considerable effect on the amount of residual monomer released from resin-based restorative materials. The amount of monomer release after topical APF application was similar to the release in the control group and was lower than the release in the NaF group. The release of monomers from the resin-based material used in the study did not approach toxic levels at the applied time intervals. The compomer released lower amounts of monomer than other resin-based materials.

**Conclusions:**

Fluoride applications do not increase monomer release from resin-based restorative materials. However, compomers should be employed by clinicians due to their lower monomer release compared to other resin restorative materials. The release of monomers from all the resin-based materials did not approach toxic levels at the applied time intervals.

**Supplementary Information:**

The online version contains supplementary material available at 10.1186/s12903-022-02698-x.

## Background

Dental caries, a biofilm-induced disease, continues to represent an important public health problem involving high costs [1, 2]. Although it can progress rapidly when left untreated, the disease is preventable, arrestable, and even reversible in the early stages through various preventive measures [3]. The most effective, simple, and economical approach in prevention is the application (by the patient or professionally) of fluoride agents daily or at regular intervals among individuals who are susceptible to caries development [4, 5]. Professionally applied topical fluorides are commercially available in gel, foam, varnish, or mouthwash forms, and their fluoride concentrations are much higher than those of self-applied fluorides [3]. These agents increase remineralization and inhibit demineralization of teeth, and also exhibit antibacterial effects [5]. Topical fluoride gels commonly used in dental practice may be acidulated or neutral. Acidulated phosphate fluoride (APF) gels, containing 1.23% fluoride ions and hydrofluoric acid, increase fluoride uptake in enamel more effectively and reduce demineralization [4, 6]. However, these agents may lead to adverse effects on resin-based composites, such as the dissolution of inorganic fillers, surface erosion, reduced surface hardness, and discoloration [4]. The New Zealand Guidelines Group, therefore, recommended using neutral gels containing 2% sodium fluoride (NaF) for patients with porcelain and composite restorations [7].

When the initial caries lesion cannot be prevented or arrested and cavitation occurs, restorative treatment plans become increasingly important in the management of carious lesions. Factors in the selection of materials to be used in restorative treatment include the individual risk of caries, the amount of tooth tissue loss, occlusal forces, and esthetic concerns [8]. Resin-based restorative materials are often preferred, especially in cases with high esthetic requirements. These restorative materials consist of organic matrices, fillers, initiators, inhibitors, and plasticizers [9]. The main components of the organic matrix are cross-linking dimethacrylate monomers, namely, bisphenol-A-glycidyl methacrylate (Bis-GMA), triethylene glycol dimethacrylate (TEGDMA), urethane dimethacrylate (UDMA), and hydroxyethyl methacrylate (HEMA) [10]. These monomers are added to the resin material to improve its mechanical properties, provide chemical stability, contribute to mimicking natural tooth color, and increase hardness, biocompatibility, and fluidity [11–13].

Monomers in resin-based materials are stabilized by forming polymer networks via photoreactions or chemical processes [11, 14]. However, complete polymerization cannot be achieved, since diffusion within the network is restricted during the crosslinking process, and the conversion of monomers into polymers is limited [10, 14]. Residual monomers may exist in the material after polymerization, or else may dissolve and be gradually released from the polymer due to several external factors, particularly in the wet oral environment [4, 15]. Monomer release from resin-based composites reduces materials’ biocompatibility [10] and exerts cytotoxic and genotoxic effects on specific cell types [16]. It also adversely affects cell homeostasis [17]. Inadequate polymerization reduces the resistance and color stability of resin-based restorative materials, thus affecting their mechanical properties [9].

Monomer release from resin-based restoratives has been evaluated in several previous studies [10, 11, 18, 19]. Nevertheless, to the best of our knowledge, no previous study has investigated whether such release is affected by fluoride applications. In order to address this deficiency, the present research investigated the effects of topical fluorides, frequently used in dentistry, on the release of residual monomers from resin-based dental materials. The null hypotheses were that fluoride applications do not affect the quantity of residual monomer release from resin-based restorative materials and that there would be no difference in monomer release between the three different resin-based materials used.

## Methods

### Materials

This research tested two types of topical fluoride gel, 1.23% APF gel (POLIMO, Imıcryl, Turkey) and 2% NaF gel (POLIMO, Imıcryl, Turkey), and three types of resin-based restorative materials, bulk-fill composite [Filtek One Bulk-Fill Restorative (FBF), 3 M ESPE, St. Paul, MN, USA], nanohybrid universal composite [Filtek Z550 (Z550), 3 M ESPE, St. Paul, MN, USA], and polyacid-modified composite resin (compomer) [Dyract XP (DXP) (Dentsply DeTrey GmbH, Konstanz, Germany]. Information regarding these materials is provided in Table 1.

**Table 1:** Materials used in the study

Resin-based materials				
Material	Type	Lot number	Ingredients	Manufacturer
Filtek One Bulk-Fill Restorative	Bulk-Fill composite	N878473	Matrix: Aromatic dimethacrylate (AUDMA), Addition-fragmentation monomers (AFM), UDMA, 1,12-Dodecanediol dimethacrylate (DDDMA) Filler: not agglomerated/not aggregated silica (20 nm), not agglomerated/not aggregated zirconia, aggregated zirconia/ silica compound, ytterbium trifluoride 58.4% volume, ~ 76.5% weight	3 M ESPE GmbH, Seefeld, Germany
Filtek Z550	Nanohybrid composite	N954930	Matrix: Bis-GMA, Bis-EMA,UDMA, PEGDMA, and TEGDMA Filler:Surface modified zirconia and silica fillers (3 μm), not agglomerated/not aggregated surface-modified silica 68% volume, 82% weight	3 M ESPE, St. Paul, MN, USA
Dyract XP	Poly acid-modified composite resin (Compomer)	2002001071	Matrix: Bisphenol-A-dimethacrylate (Bis-GMA), urethane resin, triethylene glycol dimethacrylate (TEGDMA), trimethylolpropane trimethacrylate (TMPTA), carboxylic acid-modified dimethacrylate (TCB resin), camphorquinone, dimethylamino benzoic acid ethyl ester, and butylated hydroxytoluene Filler: Strontium aluminosodium-fluoro-phosphor-silicate glass (0.8 μm 68.6% volume, 83.3% weight	Dentsply DeTrey GmbH, Konstanz, Germany

Fluoride agents					
Material	pH	Lot number	Ingredients	Manufacturer	Application procedure
Polimo 1.23% APF gel	3,5	20A249	%1,23 Acidulated Phosphate Floride	Imicryl, Konya, Turkey	60 s or up to 4 min 30 min contact time
Polimo 2% NaF gel	7	20058	2%Neutral Sodium Fluoride, Xylitol	Imicryl, Konya, Turkey	1–4 min 30 min contact time

### Specimen preparation

Ninety samples (30 for each resin-based material) were prepared by compressing the upper and lower surfaces using mylar strips between two glass surfaces to prevent oxygen inhibition and obtain a smooth surface. Each resin-based material was placed into a cylindrical plexiglass mold with a diameter of 5 mm and a depth of 2 mm. The bulk-fill composite, nanohybrid composite, and compomer were applied in single 2-mm increments and cured using a light-emitting diode (LED) unit (D-Light Pro, GC, Japan) with an irradiance of 1200 mW/cm^2^ for 20 s, as recommended by the manufacturer. A calibrated radiometer (Blast LED Light Meter, First Medica, Greensboro, NC, USA) was used to verify the irradiance of the light-curing unit.

G*Power version 3.1.9.4 software (Heinrich Heine, University of Düsseldorf, Düsseldorf, Germany) and data from previous research were used to calculate the required minimum sample size for this study [18]. In each resin-based material group (FBF, Z550, and DXP), specimens were randomly divided into three groups of 10 samples each—APF, NaF, and distilled water as the control. An alpha-type error of 0.05 and a beta power of 0.95 were established, yielding a minimum estimated sample size of 10 specimens per group.

The topical fluoride gels were applied to the sample surfaces in equal amounts for 4 min using ear sticks to simulate clinical practice, with slight stirring for approximately 1 s once every minute. The gels were kept in contact with the samples for 26 min. The surfaces of the control samples were wetted with distilled water and left standing for 30 min. Each sample was then cleaned under running tap water, after which it was immediately immersed in amber-colored glass bottles containing 1 mL of 75% ethanol solution at room temperature. The samples were stored at room temperature for four different immersion periods—10 min, 1 h, 24 h, and 30 days (the ethanol solution was replaced between the different periods).

### High-performance liquid chromatography (HPLC) analysis

Analysis was conducted using an HPLC system (Agilent Technologies, Palo Alto, CA) with a C18 reverse-phase analytical column (150 × 4.8 mm; 5 μm, ACE, Aberdeen, Scotland). The solvent consisted of 80% HPLC-grade acetonitrile (Merck KGaA, Darmstadt, Germany)/20% ultrapure water fed at a flow rate of 1 mL/min. Pure water was supplied (18.2 MWcm at 25 °C) using a Millipore purification system operated on ultrasound mode, and diluted samples were diffused with a 0.45 µm membrane filter before injection. Pure monomers for calibrating the HPLC system were obtained from Sigma-Aldrich (St. Louis, MO, USA). HEMA, TEGDMA, UDMA, and Bis-GMA were used as pure and explored as residual monomers in solvent solutions in the HPLC device. Calibration curves were constructed for each monomer. The samples were analyzed by adapting the method described by Barutçugil et al. [19]. Standard solutions were prepared in a mixture of 75% ethanol and 25% water before being preserved in a refrigerator at + 4 °C. Standard working solutions were provided separately for each monomer at different concentrations (5, 10, 25, 50, and 100 μg/mL). The amounts of monomers mixed with the samples were calculated using the standard curves obtained from the solutions. All measurements were performed in triplicate to ensure reliability, the average value for the three being used in the analysis. The correlation coefficients between the monomers and linear range mathematical equations were determined by means of linear regression analysis. Table 2 shows the regression equations, correlation coefficients, retention times, residual standard deviation percentages, limit of detection (LOD), and limit of quantitation (LOQ) of the monomer calibration curves.

**Table 2:** Calibration and sensitivity data of monomers

	Regression equation	R^2^	RT	% RSD	LOD (μg/ml)	LOQ (μg/ml)
HEMA	y = 36.398x + 6.287	0.99997	4.991	7.313	0.663	2.010
TEGDMA	y = 43.491x + 5.720	0.99999	3.150	4.903	0.372	1.127
UDMA	y = 17.161x + 3.469	0.99996	6.565	3.840	0.738	2.237
Bis-GMA	y = 35.240x + 2.079	0.99994	7.990	9.042	0.846	2.565

*y* peak area, *R*^2^ correlation coefficient, *RT* retention time, *RSD* relative standard deviations, *LOD* limit of detection, *LOQ* limit of quantification

### Statistical analysis

Statistical analysis was performed on Statistical Package for the Social Sciences software (version 25.0, SPSS Inc., Chicago, IL, USA). Data normality was checked using the Kolmogorov–Smirnov test, and continuous variables were expressed as median values (min–max). The sample groups were compared using the Mann–Whitney U and Kruskal–Wallis tests, and significance was set at *p* < 0.05. Related measurements were analyzed using the Friedman and Wilcoxon signed-rank tests. Least-squares means were compared using the Bonferroni multiple comparison procedure, a conservative process biased toward reducing false differences. Significance for comparisons of loads (three means) was set at *p* < 0.017.

## Results

Table 3 presents data on the quantities (μmol/L) of residual monomers released according to the fluoride procedures applied to the different resin-based materials (FBF, DXP, and Z550). Evaluation of monomer release based on fluoride application revealed no statistically significant difference in TEGDMA release from all resin-based materials in the NaF group (*p* > 0.05). The amounts of HEMA, UDMA, and Bis-GMA monomers released from FBF were significantly higher in the control group than in the fluoride application groups (*p* = 0.000). Significantly lower amounts of TEGDMA and HEMA monomers were released from Z550 and significantly lower amounts of UDMA and Bis-GMA monomers from DXP in the APF group (*p* = 0.000). In the NaF group, statistically higher amounts of the HEMA monomer were released from FBF. Statistically significantly lower amounts of the UDMA and Bis-GMA monomers were released from DXP (*p* = 0.000).

**Table 3:** The quantity (μmol/L) of residual monomer release according to fluoride procedures applied to different resin-based materials

Residual monomer release according to fluoride application					
Fluoride application	Material	TEGDMA	HEMA	UDMA	Bis-GMA
Control	FBF	0.235 (0.02–0.39)^a^	11.91 (0.0–58.15)^a^	10.70 (2.50–67.53)^a^	6.22 (1.99–30.76)^a^
	DXP	1.12 (0.0–2.48)^b^	3.325 (1.25–5.78)^b^	0.0 (0.0–0.0)^b^	0.82 (0.38–5.65)^b^
	Z550	0.125 (0.0–0.37)^a^	0.08 (0.0–1.07)^b^	7.42 (2.63–17.02)^b^	4.105 (1.47–11.80)^b^
		*p* = 0.000*	*p* = 0.000*	*p* = 0.000*	*p* = 0.000*
APF	FBF	0.2 (0.01–0.6)^b^	6.05 (2.64–20.17)^a^	5.56 (1.86–24.59)^a^	3.2 (1.52–12.04)^b^
	DXP	0.68 (0.29–2.03)^a^	4.89 (1.9–8.76)^b^	0.0 (0.0–0.0)^b^	1.04 (0.0–3.23)^c^
	Z550	0.086 (0.0–0.30)^b^	0.23 (0.0–0.54)^c^	7.53 (2.87–21.26)^a^	4.25 (1.72–15.07)^a^
		*p* = 0.000*	*p* = 0.000*	*p* = 0.000*	*p* = 0.000*
NaF	FBF	0.15 (0.0–0.48)	8.77 (2.10–34.86)^a^	10.16 (0.93–39.45)^a^	4.92 (0.33–18.45)^b^
	DXP	0.44 (0.17–1.08)	4.87 (2.22–9.63)^b^	0.0 (0.0–0.0)^b^	0.78 (0.46–1.97)^c^
	Z550	0.28 (0.11–3.32)	1.06 (0.17–9.99)^b^	13.87 (4.16–54.30)^a^	10.99 (2.63–33.61)^a^
		*p* = 0.056	*p* = 0.000*	*p* = 0.000*	*p* = 0.000*

*p* values are based on Kruskal–Wallis test. *p < 0.05 (bold) is significant

Lower letters indicate the difference between lines and numbers indicate the difference between columns

*FBF* Filtek Bulk Fill Composite, *DXP* Dyract XP Compomer, *Z550* 3M ESPE Z550 Nanohybrid Universal Composite, *APF* Acidulated Phosphate Fluoride Gel, *NaF* Sodium Fluoride Neutral Gel

Additional files (Additional file 1: Figure S1, Additional file 2: Figure S2, and Additional file 3: Figure S3) present the HPLC graphs of the FBF, DXP, and Z550 standards in each timeline (10 min, 1 h, 24 h, and 30 days). Table 4 presents the amounts of monomers released (μg/mL) from the resin-containing restorative materials at each immersion period (10 min, 1 h, 24 h, and 30 days) and various fluoride applications.

**Table 4:** Monomer release from resin based materials in different immersion time periods

Monomer	Material	Fluoride application	Immersion time-periods				p*
			10 min	1 h	24 h	30d	
TEGDMA	FBF	Control	0.384 (0.07–0.87)	1.083 (0.59–1.333)	0.908 (0.28–0.29)	0.978 (0.24–1.33)	0.392
		APF	1.397 (0.16–2.10)	0.699 (0.31–0.38)	0.210 (0.04–1.15)	0.699 (0.24–1.50)	0.050
		NaF	0.035 (0.00–1.47)	0.599 (0.21–1.36)	0.733 (0.42–1.68)	0.524 (0.38–0.87)	0.145
		*p*	*0.049*	*0.443*	*0.305*	*0.555*	
	DXP	Control	2.305 (0.00–3.95)	4.121 (1.75–5.03)^1^	5.064 (0.00–8.66)	3.982 (2.20–5.06)	0.095
		APF	2.375 (1.26–7.09)	1.432 (1.01–2.34)^2^	4.331 (2.62–6.95)	1.991(1.68–4.16)	0.251
		NaF	1.013 (0.59–1.61)^b^	1.257 (0.87–1.47)^2.b^	3.143 (2.17–3.77)^a^	1.816 (1.08–2.59)^b^	**0.004**
		*p*	*0.168*	***0.001***	*0.559*	*0.025*	
	Z550	Control	0.384 (0.00–1.29)	0.105 (0.00–0.45)^2^	0.419 (0.24–0.59)^2^	0.768 (0.59–0.91)^1^	**0.041**
		APF	0.105 (0.00–0.73)	0.105 (0.00–0.42)^2^	0.699 (0.42–1.01)^2^	0.349 (0.24–0.56)^2^	0.054
		NaF	1.362 (0.91–11.60)	0.768 (0.38–1.05)^1^	1.432 (0.73–1.82)^1^	0.664 (0.38–1.22)^1^	0.095
		*p*	*0.051*	***0.001***	***0.003***	***0.016***	
HEMA	FBF	Control	49.447 (9.22–223.22)	71.769 (22.67–171.74)	38.612 (24.97–160.60)	42.108 (0.00–71.85)	0.178
		APF	29.968 (2.04–77.49)	13.447 (11.14–50.72)	21.938 (17.29–53.02)	16.828 (9.99–33.04)	0.106
		NaF	33.003 (12.29–134.09)	35.808 (14.60–90.29)	53.980 (22.28–113.34)	17.865 (8.07–93.36)	0.178
		*p*	*0.285*	*0.151*	*0.222*	*0.616*	
	DXP	Control	6.378 (4.61–9.22)^1^	11.872 (8.84–13.06)	16.751 (8.84–21.90)	19.978 (12.68–22.28)	0.062
		APF	12.256 (7.68–18.83)^2.b^	17.712 (7.30–27.66)^b^	27.317 (17.67–33.81)^a^	18.403 (10.37–24.21)^b^	**0.003**
		NaF	13.524 (10.76–18.44)^2^	22.284 (18.06–28.82)	22.245 (8.45–36.88)	3.458 (1.77–4.19)	0.178
		*p*	***0.012***	*0.024*	*0.149*	*0.602*	
	Z550	Control	0.231 (0.19–3.99)^2^	0.576 (0.50–1.38)	0.307 (0.00–0.35)^2^	0.192 (0.00–1.04)	0.145
		APF	12.026 (0.58–2.08)^2^	0.615 (0.00–1.99)	1.037 (0.58–1.35)^2^	0.845 (0.00–1.38)	0.145
		NaF	26.010 (12.03–38.38)^1.a^	4.149 (1.69–19.36)^b^	4.034 (2.61–6.76)^1.b^	0.922 (0.65–1.42)^b^	**0.004**
		*p*	***0.0001***	*0.050*	***0.0001***	*0.077*	
UDMA	FBF	Control	41.697 (21.49–143.60)^a^	18.083 (9.15–99.77)^b^	17.232 (10.64–68.72)^b^	8.297 (5.32–27.87)^b^	**0.003**
		APF	19.572 (16.81–52.33)^a^	9.786 (7.02–19.57)^b^	11.275 (9.15–24.89)^b^	6.595 (4.04–12.34)^b^	**0.003**
		NaF	36.591 (19.99–83.82)^a^	23.189 (10.64–49.57)^b^	26.380 (12.13–50.21)^b^	7.446 (1.92–12.13)^b^	**0.002**
		*p*	*0.222*	*0.224*	*0.290*	*0.213*	
	DXP	Control	0.000 (0.00–0.00)	0.000 (0.00–0.00)	0.000 (0.00–0.00)	0.000 (0.00–0.00)	–
		APF	0.000 (0.00–0.00)	0.000 (0.00–0.00)	0.000 (0.00–0.00)	0.000 (0.00–0.00)	–
		NaF	0.000 (0.00–0.00)	0.000 (0.00–0.00)	0.000 (0.00–0.00)	0.000 (0.00–0.00)	–
		*p*					
	Z550	Control	28.507 (22.76–36.17)^a^	15.743 (14.47–19.99)^2.b^	16.168 (12.34–17.02)^2.b^	6.595 (5.53–7.66)^2.c^	**0.002**
		APF	34.889 (20.85–45.31)^a^	12.275 (8.51–16.93)^2.b^	17.445 (15.32–24.68)^2.b^	7.446 (6.17–11.49)^2.c^	**0.004**
		NaF	36.804 (19.36–115.52)^b^	55.950 (16.81–95.52)^1.a^	54.036 (15.96–72.33)^1.a^	13.828 (8.72–15.32)^1.c^	**0.019**
		*p*	*0.171*	***0.015***	***0.006***	***0.0001***	
Bis-GMA	FBF	Control	18.923 (10.93–60.09)^a^	10.535 (6.63–44.67)^b^	9.559 (6.63–37.46)^b^	5.072 (3.71–15.99)^b^	**0.003**
		APF	11.315 (6.83–23.41)^a^	4.292 (3.71–8.78)^b^	5.853 (5.07–12.88)^b^	3.902 (2.93–7.41)^b^	**0.004**
		NaF	22.435 (8.97–35.90)^a^	11.315 (5.27–25.17)^b^	15.412 (7.22–27.51)^b^	4.682 (0.59–9.56)^b^	**0.002**
		*p*	*0.218*	*0.153*	*0.233*	*0.215*	
	DXP	Control	0.956 (0.74–1.42)^b^	1.483 (1.01–11.02)^a^	2.165 (1.62–3.41)^a^	2.282 (1.56–3.43)^a^	**0.029**
		APF	2.146 (1.37–4.68)^a^	1.385 (1.09–4.45)^a^	2.712 (1.84–6.30)^a^	1.092 (0.00–3.00)^b^	**0.018**
		NaF	1.151 (0.98–3.51)	1.366 (1.01–1.46)	3.180 (1.95–3.84)	1.756 (0.90–2.13)	0.062
		*p*	*0.106*	*0.496*	*0.423*	*0.136*	
	Z550	Control	18.338 (13.07–23.02)^a^	7.998 (7.61–10.14)^2.b^	7.998 (7.02–8.97)^2.b^	3.512 (2.93–3.71)^2.c^	**0.002**
		APF	23.800 (13.46–29.46)^a^	7.355 (5.66–8.97)^2.b^	8.974 (7.99–13.46)^2.b^	3.902 (3.36–6.44)^2.c^	**0.004**
		NaF	31.902 (17.17–65.55)^a^	31.409 (11.32–51.11)^1.a^	31.018 (10.34–41.55)^1.a^	7.803 (5.07–9.36)^1.b^	**0.011**
		*p*	*0.057*	***0.009***	***0.004***	***0.001***	

*p* values are based on Kruskal–Wallis test and Bonferroni correction *p* < 0.05/3 = 0.017 (bolditalics) is significant; *p values based on Friedman Test *p < 0.05 (bold) is significant

Lower letters indicate the difference between lines and numbers indicate the difference between columns

*FBF* Filtek Bulk Fill Composite, *DXP* Dyract XP Compomer, *Z550* 3M ESPE Z550 Nanohybrid Universal Composite, *APF* Acidulated Phosphate Fluoride Gel, *NaF* Sodium Fluoride Neutral Gel

**Fig. 1:**
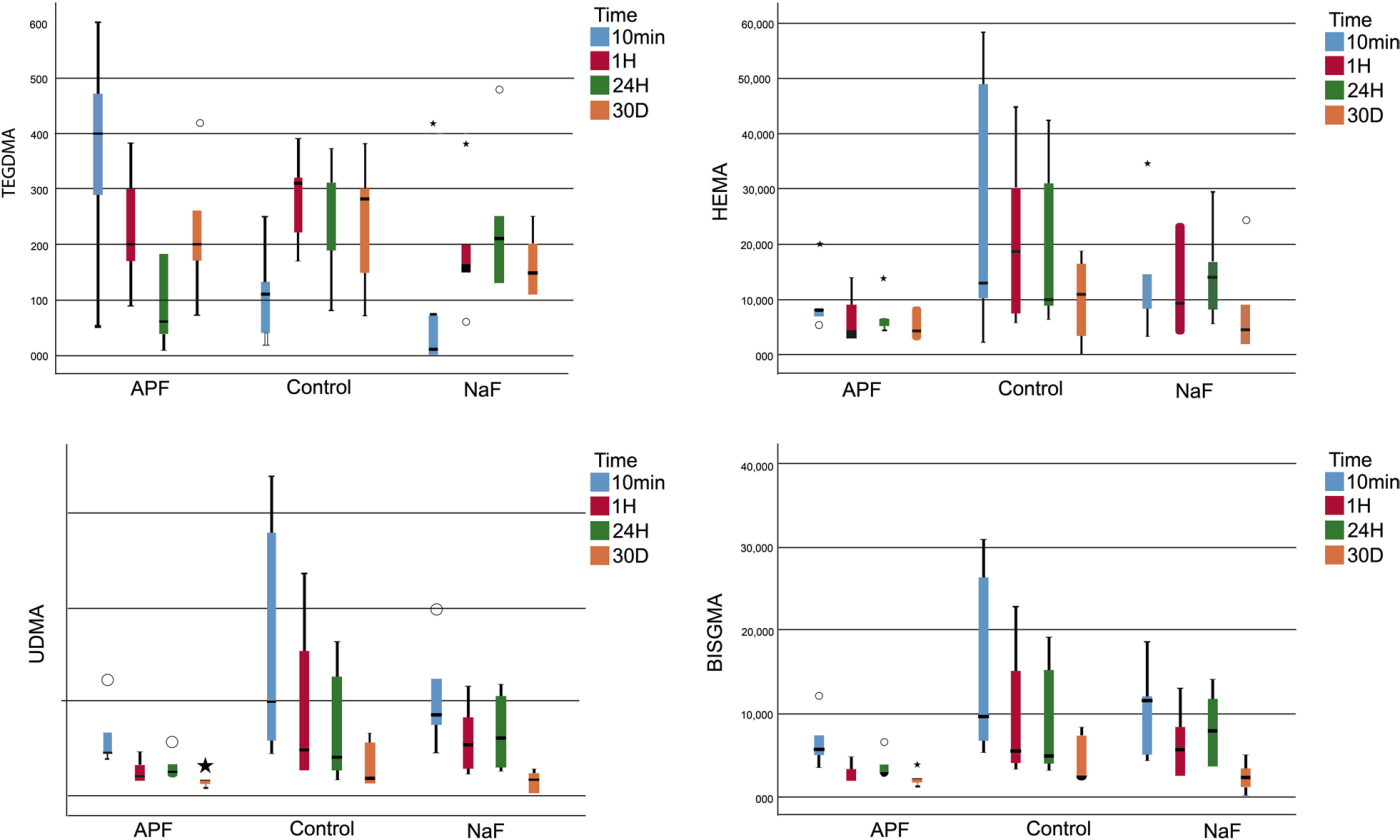
Monomer release from the Filtek One Bulk-Fill Composite after different topical fluoride applications (APF, NaF and control group) in four immersion time periods. *Bis-GMA* bisphenol-A-glycidyl methacrylate, *TEGDMA* triethylene glycol dimethacrylate, *UDMA* urethane dimethacrylate, *HEMA* hydroxyethyl methacrylate, *APF* Acidulated Phosphate Fluoride Gel, *NaF* Sodium Fluoride Neutral Gel

For FBF, no significant difference in the release of TEGDMA, HEMA, UDMA, and Bis-GMA monomers was detected at any immersion period following fluoride application (*p* > 0.017). No significant difference was also observed in the release of TEGDMA and HEMA monomers in terms of the difference between the four immersion periods following fluoride applications (*p > 0.05, Fig. 1). However, a significant difference in release was determined among the four immersion periods due to the time-dependent decrease in the released UDMA and Bis-GMA monomers (*p < 0.05, Fig. 1).

**Fig. 2:**
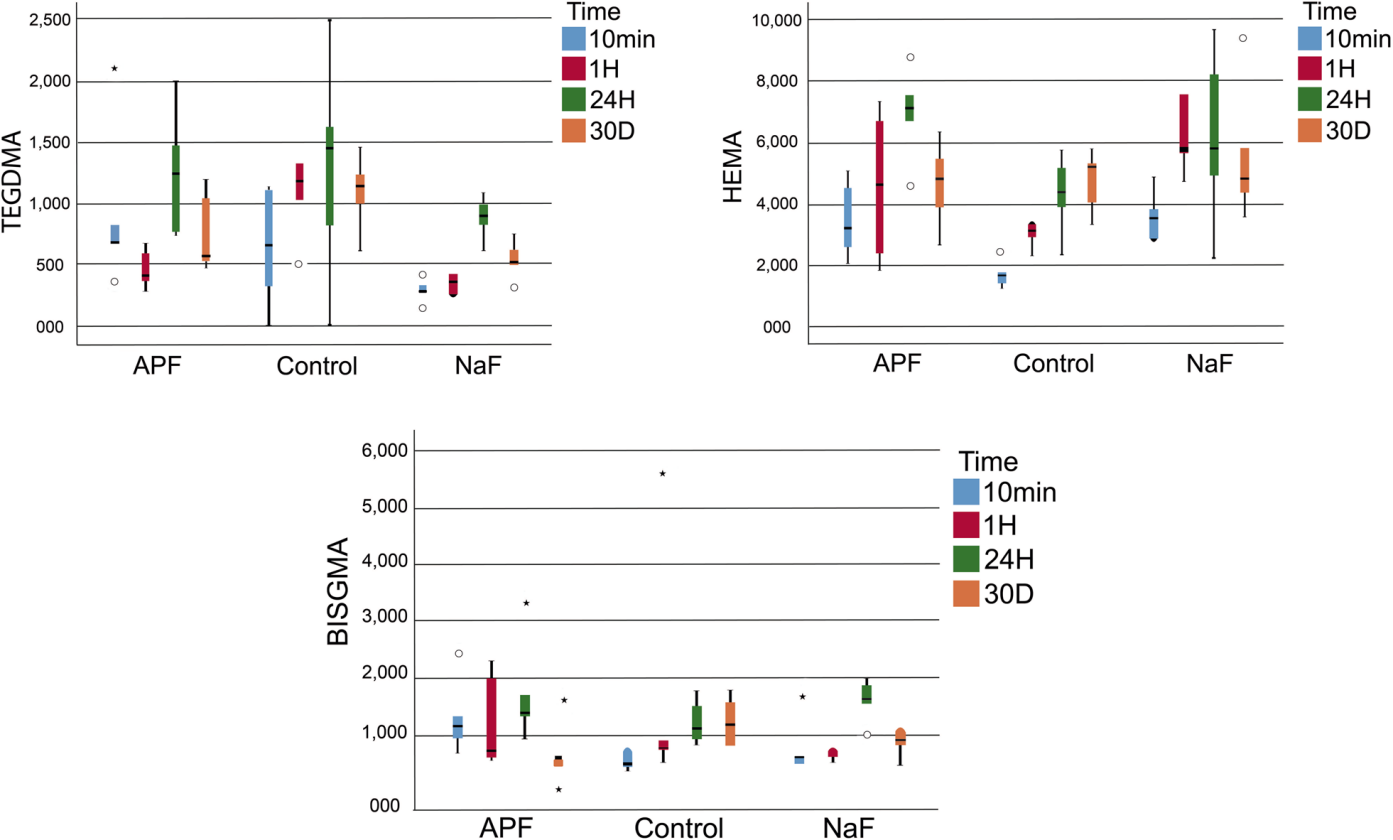
Monomer release from the Dyract XP Compomer after different topical fluoride applications (APF, NaF and control group) in four immersion time periods. *Bis-GMA* bisphenol-A-glycidyl methacrylate, *TEGDMA* triethylene glycol dimethacrylate, *HEMA* hydroxyethyl methacrylate, *APF* Acidulated Phosphate Fluoride Gel, *NaF* Sodium Fluoride Neutral Gel

For DXP, the release of the TEGDMA monomer at 1 h was significantly higher in the control group than in the fluoride-applied groups (*p* = 0.001)*.* Subgroup analyses of the NaF group indicated that the statistical difference in the time-dependent changes in TEGDMA release derived from the higher release of the monomer at 24 h than in the other periods (*p = 0.004, Fig. 2). The release of the HEMA monomer at 10 min was significantly lower in the control group than in the fluoride-applied groups (*p* = 0.012). Subgroup analyses of the APF group showed that the statistical difference in the time-dependent changes in HEMA monomer release was due to the higher release of the monomer at 24 h than in the other periods (*p = 0.003, Fig. 2). The UDMA monomer was either not eluated or else was released at an undetectable level. Subgroup analyses of the control group revealed that the statistical difference in the time-dependent changes in Bis-GMA monomer release derived from the lower release at 10 min than in the other periods (*p = 0.029, Fig. 2). Additionally, the statistical difference in the time-dependent changes in the Bis-GMA monomer release in the APF subgroup resulted from the lower release of the monomer on the 30th day compared with the other periods (*p = 0.018, Fig. 2).

**Fig. 3:**
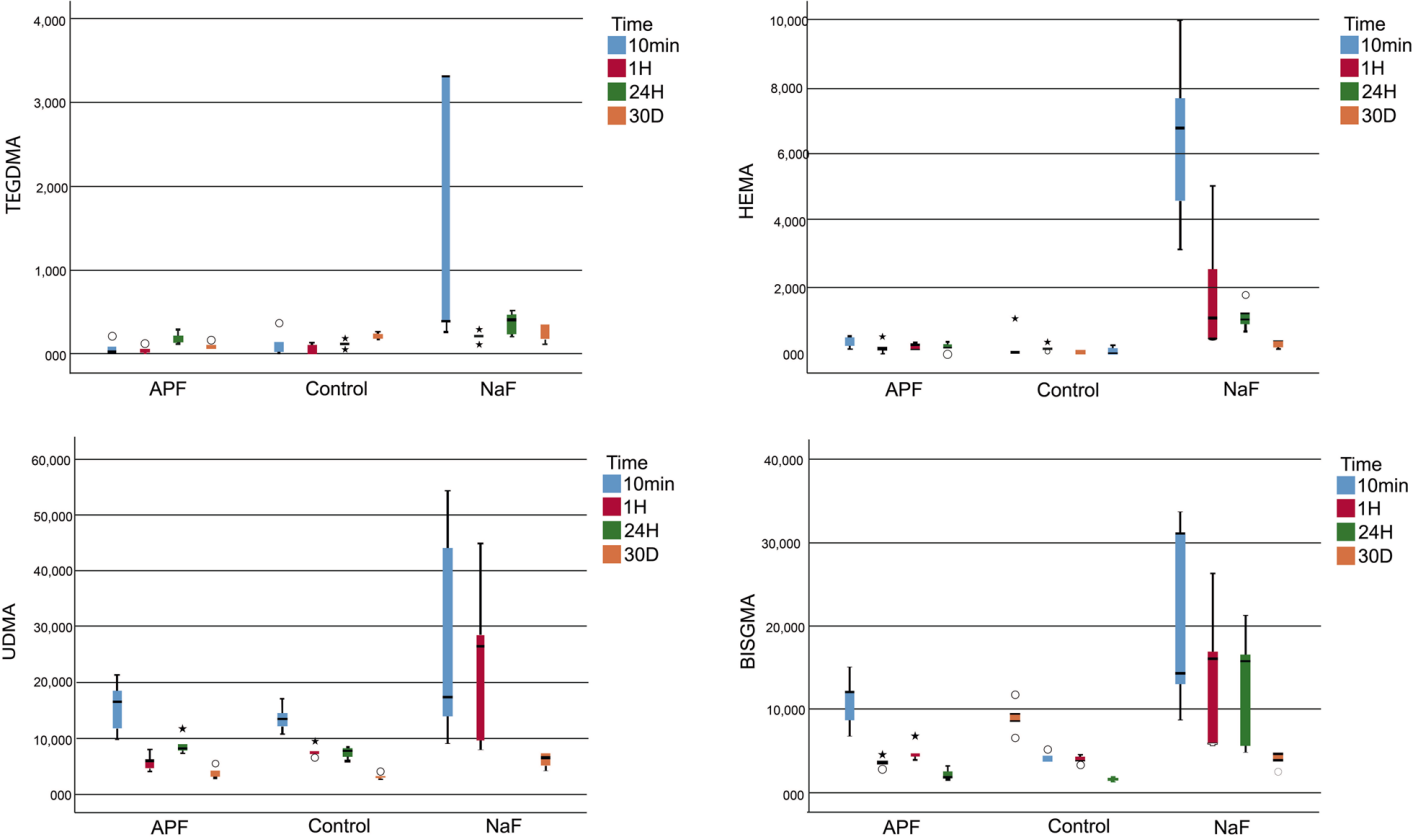
Monomer release from the Z550 Nanohybrid Universal Composite after different topical fluoride applications (APF, NaF and control group) in four immersion time periods. *Bis-GMA* bisphenol-A-glycidyl methacrylate, *TEGDMA* triethylene glycol dimethacrylate, *UDMA* urethane dimethacrylate, *HEMA* hydroxyethyl methacrylate, *APF* Acidulated Phosphate Fluoride Gel, *NaF* Sodium Fluoride Neutral

For Z550, TEGDMA monomer release was significantly higher in the NaF group at 1 h and 24 h (*p* = 0.001 and *p* = 0.003, respectively), but was significantly lower on the 30th day in the APF group (*p* = 0.016). The release of the HEMA monomer at 10 min and 24 h was significantly higher in the NaF group (*p* = 0.0001). Subgroup analyses of the NaF group showed that the statistical difference in the time-dependent changes in HEMA monomer release was due to the greater release of the monomer at 10 min (*p = 0.004, Fig. 3). The release of the UDMA monomer at 1 h and 24 h and 30 days was significantly higher in the NaF group than in the other groups (*p* = 0.015, *p* = 0.006, and *p* = 0.0001, respectively). The time-dependent changes in UDMA monomer release pointed to a statistically significant decrease in release over time in the control and APF groups (*p = 0.002 and *p = 0.004, respectively; Fig. 3). In the NaF group, monomer release was statistically higher at 1 h and 24 h (*p = 0.019, Fig. 3). The release of the Bis-GMA monomer at 1 h, 24 h, and 30 days was significantly higher in the NaF group than in the other groups (*p* = 0.009, *p* = 0.004, and *p* = 0.001, respectively). The time-dependent changes in Bis-GMA monomer release revealed a statistically significant decrease over time in all the study groups (*p = 0.002, *p = 0.004, and *p = 0.011, respectively; Fig. 3).

## Discussion

Based on the study findings, although the monomer release from the resin-based materials after fluoride applications did not generally exhibit significant differences with the control group, higher or rarely lower monomer releases were occasionally observed compared to the control group. The null hypothesis, that fluoride applications do not affect the quantity of residual monomer release from resin-based restorative materials, was thus partially confirmed based on the absence of effects of fluoride applications (APF and NaF) on monomer release from the resins in some of the immersion periods.

Clinicians decide on the material to be used in restorative dentistry depending on the patient's age, dentition type, cavity structure, depth, occlusal stress, and esthetic needs [20]. While nanohybrid composites are preferred in high-stress areas due to their high inorganic content and advanced mechanical/esthetic properties [21], bulk-fill composites are frequently preferred in deep cavities because they allow 4–6 mm spacing and reduce the number of clinical steps required [22, 23]. Compomers are the most widely used materials for the restoration of primary teeth. This material leads to the release of fluoride by delayed acid–base reaction of glass ionomer hydrogel formation [24]. Nanohybrid composite (Z550), compomer (DXP), and bulk-fill composite (FBF) were used in the present study.

The placement of conventional composite resins using incremental techniques is recommended in order to permit improved penetration of curing light throughout a material [25]. This protocol reduces polymerization stress and ensures homogeneous conversion [26]. Bulk fill composites, on the other hand, allow the cavity to be filled in a single step [27]. However, conflicting results have been reported in the literature regarding whether the recommended layer thickness for bulk-fill composites is sufficient for polymerization [28]. Kılıç et al. [29] compared the quantity of residual monomers leached from bulk-fill composites with different compositions polymerized at varying layer thicknesses. Those authors concluded that using bulk-fill composites in the form of 2 + 2 mm layers instead of a single 4 mm thick layer may reduce residual monomer release from the materials. In the light of these findings, all restorative materials in the current research were prepared at a layer thickness of 2 mm to ensure standardization.

Various approaches, such as HPLC, gas–liquid chromatography [30], and mass spectrometry [31], are used to determine the amount of residual monomers released from materials. However, chromatographic techniques are the most appropriate methods for analyzing the release from resin-based materials. HPLC, used in this study to evaluate the release of residual monomers from resin-based materials after fluoride application, is the most widely employed measurement method [10, 11, 14, 18, 19]. This is because it enables the dissolution of monomers in the mobile phase during the separation process, thus yielding advantages such as easier control over elution, reproducibility, reliability, rapidity, economy, selectivity in results, and applicability to temperature-sensitive substances [32].

The degradation of composite resins in the oral cavity is due to enzymatic reactions in saliva, acidic conditions, and erosive factors caused by food and drink [33]. Organic solvents (e.g., ethanol and methanol) or mixtures of these solvents with water should be employed in order to mimic these conditions [33, 34]. In this context, various storage solutions are used to investigate the cytotoxicity of dental materials, such as distilled water, physiological saline, ethanol, methanol, acetone- and ethanol-added saline, artificial saliva, and cell culture medium [35]. Ethanol penetrates the polymer network of resin-based restorative materials, widens the gaps between polymer chains, and facilitates monomer release [18, 36, 37]. These properties have led to its being used as a storage medium in many studies evaluating residual monomer release [10, 19, 29, 38]. A mixed solution of 75% ethanol and 25% water, recommended by the United States Food and Drug Administration as a liquid that mimics the food–oral cavity relationship, was used as the storage medium in the present study [35, 39].

Numerous studies have investigated the release of monomers from resin-based materials. Material type [36, 40], layer thickness [29], light-curing units [33], polymerization time [11], finishing and polishing procedures [18], bleaching [10], and chewing [41] have been described as capable of altering the quantity of residual monomer release from resin-based restorative materials. However the effects of fluoride application on the concentration of residual monomer release from restorative materials have remained unclear. The present study thus sought to fill this gap in the literature.

It is generally recommended that topical fluoride agents be applied every six months among individuals prone to caries development [4]. However, whether resin composites exposed to these agents cause surface degradation has been a matter of concern. Researchers have investigated whether these agents, which are applied for protective purposes in dentistry, cause surface changes such as surface roughness, microhardness, and elemental changes in resin-based composites [4, 42–44]. Since APF gels contain strong acids, the composition and surface integrity of composites and other glass-containing restoratives can change significantly. Increased filler dissolution may result in raised exposure of the organic matrix and consequently an accelerated hydrolytic effect. During this process, fluoride ion is involved in the depolymerization reaction of the matrix-filler interface [28]. Another reason is thought to be that low pH agents affect the sorption, solubility, and surface degradation of the resin composite [42]. However, Yeh et al. [4] reported that the main factor responsible for surface deterioration is related to the amount of hydrofluoric acid (HF), rather than the pH or viscosity of the fluoride gels. The H^+^ and F^−^ ions in the APF gels (chemical formula FH_3_NaO_4_P) form a covalent bond and cause the formation of HF, which accelerate the decomposition of the silica fillers of the restorative materials [45, 46]. However, studies have reported that neutral fluoride gels and APF gels containing thixotropic components do not give rise to this problem [4]. Thixotropic components comprise magnesium aluminum silicate (MAS) clay and silica dioxide powder. MAS is a structure with negatively and positively charged layers that form three-dimensional networks resulting from the attraction between these two opposite charges. Researchers have hypothesized that F − ions are attracted to the positively charged layers of the MAS in APF gels containing thixotropic components, while H + ions are attracted to the negatively charged layers, thus preventing the formation of HF [4]. The amount of deterioration caused by APF gels on the surface of resin composites has thus been reported to be reduced by the MAS component. In the present study, monomer release after NaF and APF (containing thixotropic components) application was largely similar to that in the control group, and even lower in some cases. We think that the effect of topical fluorides on monomer release is related to the mechanism described above. In other words, the low HF ratio causes less surface deformation, and the resin matrix remains more stable. In this context, monomer release similar to that the control group in the fluoride treatment groups may be regarded as an expected result in the current study.

In terms of monomer releases among the materials, UDMA and Bis-GMA releases were higher in Z550 than in FBF and DXP after both topical fluoride applications. We attributed this higher release to the greater inorganic content in Z550 than in the other two resin-based materials. With the dissolution of larger inorganic content, the amount of degradation on the material surface may increase, resulting in higher monomer release from the resin matrix [21]. In the light of this result, the second null hypothesis, that there would be no difference in monomer release between the three different resin-based materials, was rejected.

Since the release of monomers (not at toxic levels) after fluoride application varies depending on the type of resin material used, the selection of the most appropriate material in clinical practice needs to be considered. The rates at which the monomers investigated by Kılıç et al. [29] were released followed the order UDMA > Bis-GMA > TEGDMA > HEMA. Consistent with that study, the most extensively released monomer in the current research was UDMA, followed by HEMA, Bis-GMA, and TEGDMA. Similarly to Sajani et al.’s study [47], the total elution of Bis-GMA was higher than that of TEGDMA in all the materials used in our research. This difference may potentially derive from the low double bond conversion and differences in the chemical properties of Bis-GMA and considerable release of this when ethanol is used for sample storage. It may also be due to variations in the materials and working methods used.

Although considerable progress has been made in developing resin-based restorative materials, residual monomer release after polymerization reaction remains a problem. The release of these monomers can lead to several hazards to human health [10]. In addition to exerting cytotoxic, genotoxic, mutagenic, and estrogenic effects, they can cause local and systemic allergic reactions, soft tissue irritation, and significant proliferation of cariogenic microorganisms [9, 48]. Cell culture studies have shown that these monomers can also cause apoptosis by increasing the amount of reactive oxygen and oxidative stress in a cell [17]. Most studies on the effects of composite components on human health have used different techniques and different primary and permanent cells [48, 49]. These may result in significant limitations when comparing cytotoxicity data. Based on the limited available data, toxicity among monomers is ranked from most to least toxic as follows: Bis-GMA > UDMA > TEGDMA > HEMA [49].

Researchers have observed 40% inhibition in dental pulp cells exposed to Bis-GMA at a concentration of 0.075 mmol/L [48]. Reichl et al. [50] showed that the half-maximum effective concentration (EC_50_, causing a 50% reduction in cell viability) in human gingival fibroblasts is 0.087 mmol/L. Bis-GMA, reported as the most toxic monomer [48], did not reach the concentrations reported to affect cell viability in the material groups in the present study. DXP exhibited the lowest Bis-GMA release among the resin-based restorative materials under fluoride treatments. UDMA, which increases the degree of monomer conversion and the depth of polymerization in composite resins, degrades more quickly than solid monomers, such as Bis-GMA, because it produces a heterogeneous polymer network owing to its flexibility [51]. This may explain the high UDMA release from two resin-based restorative materials (FBF and Z550) used in our study. The EC_50_ value of this monomer in human gingival fibroblasts is 0.106 mmol [50]. In the present research, the UDMA monomer released from the materials after different fluoride applications was in the µmol/L range, 1000 times lower than the toxic doses reported.

TEGDMA, a hydrophilic monomer, can react with intracellular molecules by penetrating the membranes of oral tissues [52]. This monomer induces intracellular glutathione reduction and causes severe cytotoxicity in periodontal ligament fibroblast cultures [53]. The toxic dose of TEGDMA released from composite resins in human oral mucous membrane cells is 3.7 mmol/L [50]. In the present study, the amount of monomer released did not approach the toxic value at any of the time periods adopted. Indeed, topical fluoride applications caused a decrease in TEGDMA release, except for NaF application to Z550. According to the material safety data sheet, information should be provided only on the main constituents of the materials above 1%. However, as Yılmaz and Gül [54] stated in their study, the release of monomers not specified in the manufacturer's instructions was observed, albeit in small amounts. Similarly to that study, monomer releases from the resin based materials used that were not specified by the manufacturer were observed in the present research. The FBF manufacturer emphasizes that the Bis-GMA monomer has been replaced by a dimethacrylate that does not use Bisphenol A in its synthesis. In addition, HEMA and TEGDMA are not mentioned in their instructions. However, the release of all these monomers has been demonstrated in some studies [10, 33]. The results of the present study showed that TEGDMA, HEMA, UDMA, and Bis-GMA monomers were released from FBF, findings consistent with the previous literature. Similarly, although the manufacturers did not mention the HEMA content in Z550 and DXP, release of this monomer from these materials was detected in the current study as well as by previous researchers [10, 11, 29]. These releases may be due to the manufacturers not mentioning concentrations below 1%.

HEMA is a water-soluble monomer incorporated into the structure of composite resins because of its hydrophilic nature. Its small size, low molecular weight, inherent flexibility, and self-cross-linking ability result in substantial release [55]. The toxic dose of HEMA for human gingival fibroblasts is 3 mmol/L, and that for human pulp fibroblast cells is 10 mmol/L. In the present study, the release of this monomer after different fluoride treatments was at the μmol/L level, but did not approach reported toxic doses in any of the time intervals applied.

Various opinions have been expressed concerning the time required for the complete release of unreacted monomers. For instance, Alshali et al. [36] asserted that monomer release from samples can last for weeks or even months, but that the highest release occurs in the first days after polymerization. Considering the time-dependent changes that may occur in monomer release, the present study evaluated this phenomenon over a period from 10 min to 30 days. The time-dependent change in monomer release in most groups exhibited no significant difference in the adopted periods. However, a significant decrease in monomer release was observed over time in some groups.

There are a number of limitations to this in-vitro study. First, it evaluated the release of only a few residual monomers (TEGDMA, HEMA, UDMA, and Bis-GMA), thus providing only narrow information on the issues of interest. Second, the samples were kept in an ethanol-distilled water storage solution, a liquid that imitates the relationship between food and the oral environment. However, the mechanical and chemical effects occurring in the oral environment could not be simulated. Third, HPLC analysis results were not supported by infrared spectroscopy analysis, and unreacted monomers could not therefore be detected. The final limitation is that only the effect of only gel-formed fluoride preparations on monomer release was investigated.

## Conclusion

Based on the findings and the limitations of this in-vitro evaluation, the results may be summarized as follows: Monomer release from resin-based restorative materials does not increase over time after fluoride applications. Although the amount of monomer release after topical APF application was largely similar to that observed for the control group, it was lower than the degree of release occurring after NaF application. The release of monomers in all resin-based materials did not approach toxic levels at any of the time intervals applied. However, the compomer released lower amounts of monomers than those released by the bulk-fill composite and nanohybrid universal composite. Dentists should consider the amounts of monomer released when selecting restorative agents for tooth restorations in the clinical setting. Further high-quality randomized controlled clinical or animal trials are now required to evaluate the effect of fluoride applications on monomer release from resin-based materials.

## Supplementary Information

**Additional file 1: **HPLC graphs of standards of FBF in each timeline.

**Additional file 2: **HPLC graphs of standards of DXP in each timeline.

**Additional file 3: **HPLC graphs of standards of Z550 in each timeline.

## Data Availability

The datasets and materials used or analysed during the current study are available from the corresponding author on reasonable request.

## Abbreviations

APF: Acidulated phosphate fluoride
NaF: Sodium fluoride
Bis-GMA: Bisphenol-A-glycidyl methacrylate
TEGDMA: Triethylene glycol dimethacrylate
UDMA: Urethane dimethacrylate
HEMA: Hydroxyethyl methacrylate
FBF: Filtek One Bulk-Fill Restorative
Z550: Filtek Z550
DXP: Dyract XP
LED: Light emitting diode
HPLC: High-performance liquid chromatography
LOD: Limit of detection
LOQ: Limit of quantitation
MAS: Magnesium aluminum silicate
