# Supplementary figures and images for: Effect of topical fluoride applications on residual monomer release from resin-based restorative materials
Source: BMC Oral Health. 2023 Jan 2;23:1. doi: 10.1186/s12903-022-02698-x (PMC9808933; doi:10.1186/s12903-022-02698-x)

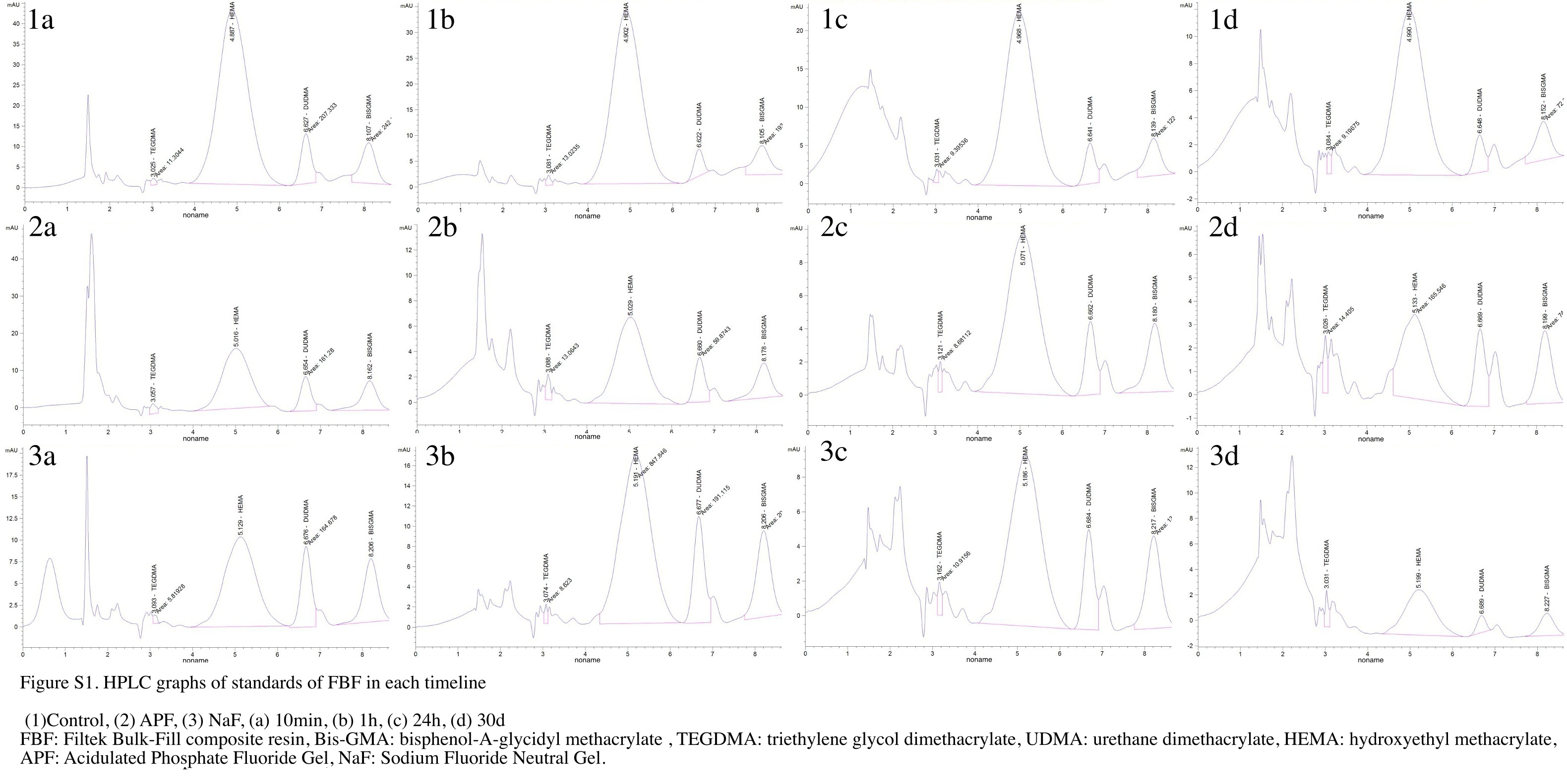

Supplement: Supplementary file 1 — Additional file 1: HPLC graphs of standards of FBF in each timeline. [file 12903_2022_2698_MOESM1_ESM.jpg]

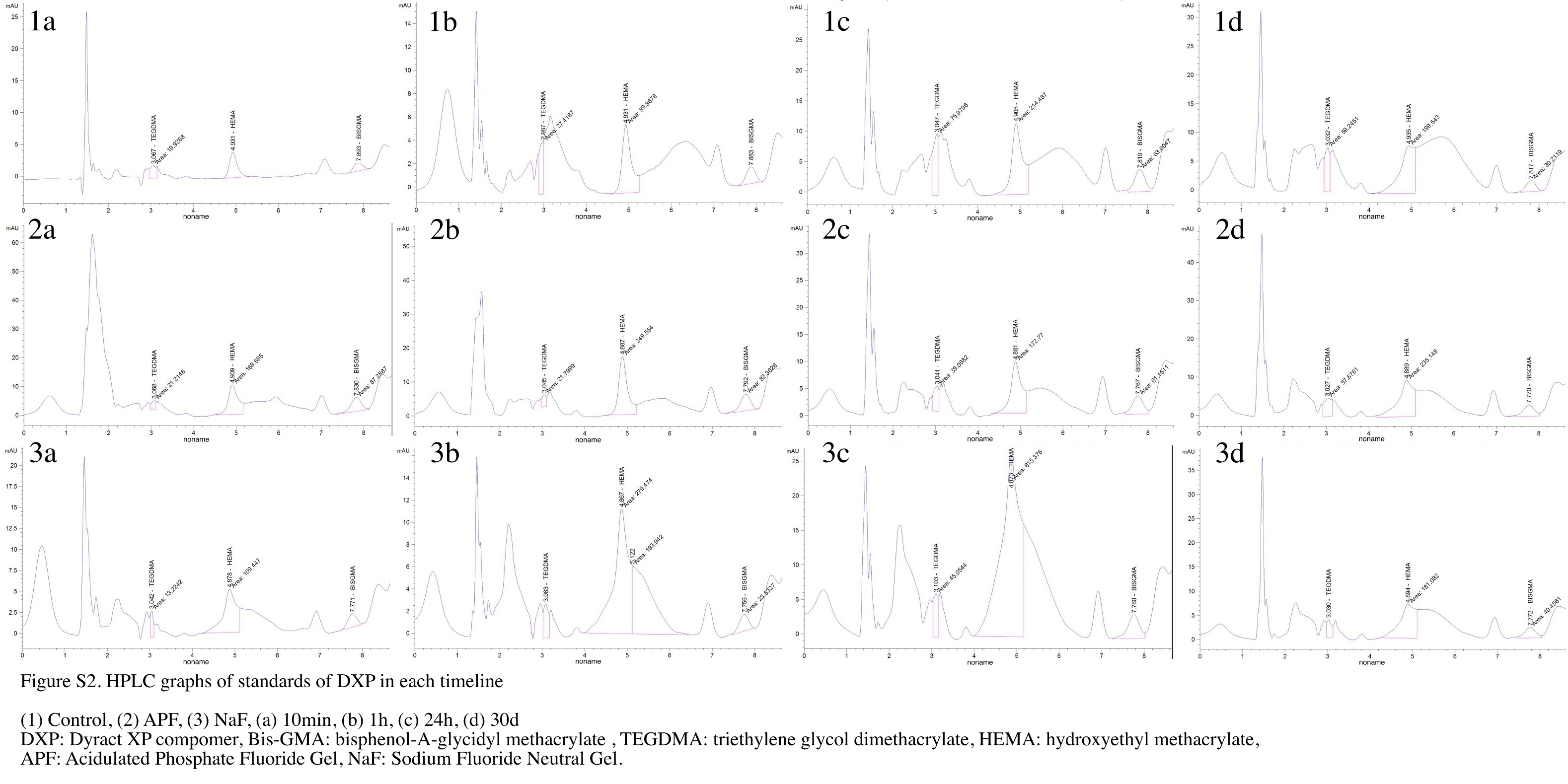

Supplement: Supplementary file 2 — Additional file 2: HPLC graphs of standards of DXP in each timeline. [file 12903_2022_2698_MOESM2_ESM.jpg]

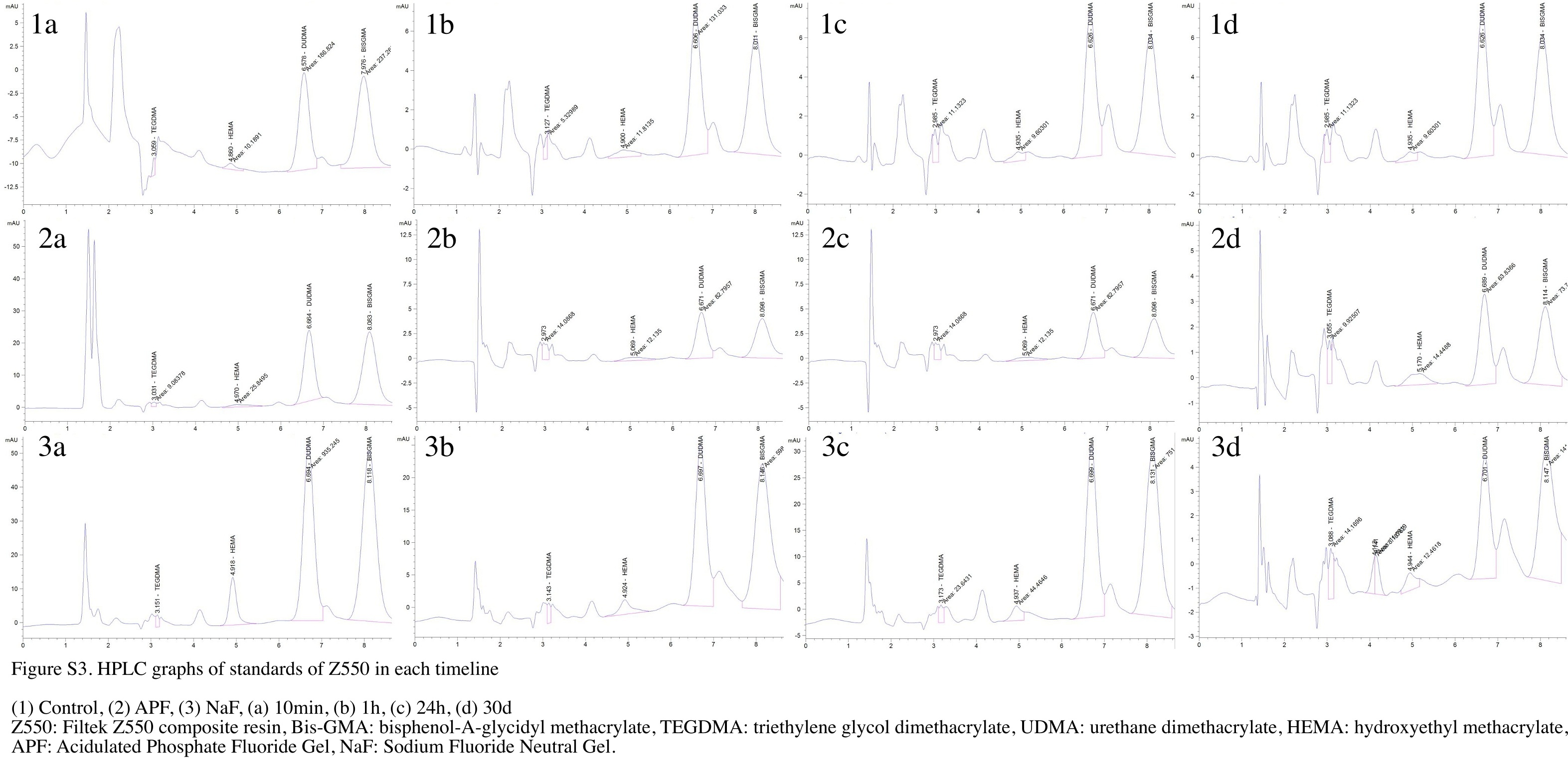

Supplement: Supplementary file 3 — Additional file 3: HPLC graphs of standards of Z550 in each timeline. [file 12903_2022_2698_MOESM3_ESM.jpg]
